# Birth Origin Differentially Affects Depressive-Like Behaviours: Are Captive-Born Cynomolgus Monkeys More Vulnerable to Depression than Their Wild-Born Counterparts?

**DOI:** 10.1371/journal.pone.0067711

**Published:** 2013-07-04

**Authors:** Sandrine MJ. Camus, Céline Rochais, Catherine Blois-Heulin, Qin Li, Martine Hausberger, Erwan Bezard

**Affiliations:** 1 Université Bordeaux, Institut des Maladies Neurodégénératives, UMR 5293, Bordeaux, France; 2 CNRS, Institut des Maladies Neurodégénératives, UMR 5293, Bordeaux, France; 3 UMR 6552 CNRS Ethologie animale et humaine, Université de Rennes 1, Station Biologique Paimpont, France; 4 Motac France, Floirac, France; 5 UMR 6552 CNRS Ethologie animale et humaine, Université de Rennes 1, Rennes, France; INSERM/CNRS, France

## Abstract

**Background:**

Adverse early-life experience might lead to the expression of abnormal behaviours in animals and the predisposition to psychiatric disorder (*e.g.* major depressive disorder) in Humans. Common breeding processes employ weaning and housing conditions different from what happens in the wild.

**Methods:**

The present study, therefore, investigated whether birth origin impacts the possible existence of spontaneous atypical/abnormal behaviours displayed by 40 captive-born and 40 wild-born socially-housed cynomolgus macaques in farming conditions using an unbiased ethological scan-sampling analysis followed by multifactorial correspondence and hierarchical clustering analyses.

**Results:**

We identified 10 distinct profiles (groups A to J) that significantly differed on several behaviours, body postures, body orientations, distances between individuals and locations in the cage. Data suggest that 4 captive-born and 1 wild-born animals (groups G and J) present depressive-like symptoms, unnatural early life events thereby increasing the risk of developing pathological symptoms. General differences were also highlighted between the captive- and wild-born populations, implying the expression of differential coping mechanisms in response to the same captive environment.

**Conclusions:**

Birth origin thus impacts the development of atypical ethologically-defined behavioural profiles, reminiscent of certain depressive-like symptoms. The use of unbiased behavioural observations might allow the identification of animal models of human mental/behavioural disorders and their most appropriate control groups.

## Introduction

Early-life experience plays a key role in the personality development in general and in the predisposition to mental disorders [1]–[3]. The pioneering work of Harlow (on non-human primates - NHPs) and Bowlby (on orphan children) in the 1960’s were based on the hypothesis that early life adverse events, such as maternal separations or death of a parent, increased the expression of atypical behaviours and the risk of developing psychiatric symptoms [4]–[7]. Since then, accumulating preclinical evidence has supported these assumptions. For instance, the age of the individual when submitted to social separations [8], the number of handling or medical procedures experienced [9] or the early rearing and housing conditions [9]–[11] have been shown to influence the expression and frequency of atypical behaviours such as stereotypic behaviours (SB, defined in [12]) in NHPs. Behavioural time budgeting (*i.e.* the relative distribution of behaviours expressed during the observational sessions) is a powerful indicator of animals’ well-being since it can be altered qualitatively (*i.e.* expression of unusual behaviours) or quantitatively (*i.e.* changes in the frequencies of usual behaviours) in captive individuals [13]–[16]. With time budgeting, we identified in our previous study spontaneous atypical behavioural profiles reminiscent of depressive-like (*e.g.* inactivity, low level of exploration, long time spend facing the wall, mimicking decrease of interest in usual activities, psychomotor slowdown, and energy loss [17]) and anxiety-like (*e.g.* displacement behaviours, aggressiveness, and a low level of inactivity mimicking anxiety, irritability and restlessness [17]) symptoms among captive-born single-housed cynomolgus monkeys [18]. Identifying such profiles in social groups, a more naturalistic housing condition for macaques, would increase the face validity of these promising ethological models. A few studies have suggested origin as a risk factor for the development of atypical behaviours, such as more repetitive behaviours displayed by captive-bred animals compared to their wild-caught congeners [19] or a different level of reactivity and fearfulness in captive Chinese-Indian hybrid rhesus macaques compared to their pure Indian peers [20], [21]. Since breeding processes for NHPs usually imply a weaning around 5-months of age followed by a peer-rearing until 3-years old, rearing conditions in captivity are quite different from the wild where infants are weaned around the age of 1 year-old and continue to interact regularly with their mother afterwards and with the other adults in the troop [22]–[24].

The question whether captive-born monkeys express more atypical behavioural profiles than wild-born individuals therefore arises. To follow on our previous study [18] and by availing of NHPs breeding farms, we thus investigated (i) whether animals from different origin (*i.e.* captive-born vs wild-born socially-housed farm animals) expressed distinct behavioural time budgets and (ii) whether similar spontaneous atypical profiles reminiscent of human depressive symptoms could be identified in these 2 populations. We adapted to social groups our previously established observational and analysis methodological protocols [18]. We hypothesised that the differential early life environment might lead to distinct coping mechanisms in captive- and wild-born monkeys, namely captive-born individuals might be more vulnerable to depressive-like states than wild-born based upon the cumulative stress hypothesis of depression [25], [26].

## Materials and Methods

### Animals and Housing Conditions

Eighty adult (mean age: 5.9±0.1 years old) female cynomolgus monkeys (*Macaca fascicularis*) were studied in a breeding farm (the Fangcheng Gang Spring Biological Technology Development Corporation, Guangxi province, Popular Republic of China). Monkeys from both origin (n_captive-born_ = 40; n_wild-born_ = 40) were housed indoors in one-male, multi-female (17 to 27 females) cages (L3.50 m×W7 m×H3 m) containing several sitting benches and one swing (see **Figure S1** for a schematic view of a regular social-housing cage). Animals were fed SAUE Ltd Old World Monkey pellets (Beijing, China) twice, and fruit once daily (regular diet). Water was provided *ad libitum*. Half of the cages’ roof was wire-meshed, therefore conferring natural outside light cycle.

Animals from 8 different cages were included in the study. The four captive-born cages were adjacent to each other, as were the four wild-born cages. They could not see each other because the walls were made of opaque concrete but they had visual contacts with the opposite cages from the same origin and not included in our study. All 8 groups were housed in the same building, to prevent bias due to husbandry habits or caretaker behaviour, and could therefore have auditory and olfactory contacts with monkeys from other groups. These 8 social groups had remained stable for at least 9 months prior to the beginning of the observations. A clear stable hierarchy was thus established. Ten animals per cage were “randomly” selected from a list prior to any interaction with them. The included animals were observed in their usual home cage, no social reorganization was performed. Forty individuals were born in this facility (“captive-born group”). Their early life experience involved the usual breeding process: a weaning from their social group around 6-months old followed by a peer-housing until 3 years of age. They were then assigned to a one-male, multi-female breeding group. The other forty females were born in Cambodia (“wild-born group”), and brought to the facility in 2009 (their age ranging from 4 to 8 years old at that time). Their wild early life environment was unknown (*e.g.* gender-ratio, group size or hierarchical rank). Females from these two origins were never mixed within the groups. The male in the cage was from the same origin as the females. The age and number of parturitions of the wild-born females were estimated by the veterinarian using dentition state, mammary and abdominal slackening. These data are therefore less accurate than the ones for the captive-born individuals. In order to facilitate the identification of the females (by the observer) and using gentle restraint, coloured medals were added to the monkeys’ collar and their back hair was shaven with distinct signs at least 72 hours prior to the beginning of the observations. The animals were therefore easily recognizable by the observer during the scan sampling observations.

### Ethics Statement

The institutional animal care and use committee of the Institute of Lab Animal Science of Chinese Academy of Medical Science approved this study. The housing conditions were in compliance with the guidelines of the Haikou Forestry Office (Hannan Province, People’s Republic of China). Such conditions correspond to standard practices in operation in breeding facilities providing macaques to the whole Japanese, American and European toxicology industry and research laboratories. All procedures described were observational and did not involved physical manipulation (except for the collaring phase performed by trained animal caretakers), nor changes in their environments or diet. Veterinarians skilled in the healthcare and maintenance of non-human primates supervised animal care. No animal was harmed or killed in the course of the experiments.

### Behavioural Assessment

One day before the beginning of the observations, the observer sat in front of the cage (at the same location as during the observations) at the same time as during the observational sessions (*i.e.* 9–11 am and 3–5 pm) to allow monkeys to habituate to her presence. Macaque behaviour was scored and recorded live by two trained observers (SC, CR; inter-observer reliability: Spearman rank order correlation R = 0.86) outside the feeding and cleaning times, in a randomized order at two time points (morning and afternoon) on 6 consecutive days. The observers were sitting 1 meter away from the front of the cages. We used a scan-sampling method, appropriate for time budgeting [27], in which behavioural parameters were assessed every 6 minutes during 2-hour sessions, resulting in 240 scans per individual. We focused on behavioural profiles rather than single items. Two repertoires, adapted from [18] and completed with any additional items observed during our observations, were used: one reporting the interaction with the environment (**Table S1**) and one describing the position within the environment (**Table S2**). We investigated the percentages of occurrence of each item with regard to the 240 scans (behavioural and postural time budgets as well as location, body orientation and inter-peer distance profiles) and the behavioural diversity (the number of different behaviours expressed during the 240 scans).

### Factor Analyses

Wild- and captive-born populations were analysed separately as the aim of the study was to investigate the differential presence of atypical profiles among both populations.

As data were not normally distributed, they were submitted to multifactorial correspondence analyses (MCA; SPAD^©^ 7.4, Coheris) that uses chi-square criterion to assess differences and similarities between frequencies of qualitative variables. Active variables are placed in a multidimensional cloud in which two items are at a short distance if they show similar proportions in the same individuals and conversely they are distant if expressed by different individuals. The same process is then repeated with individuals. Two individuals are close if they share similar behavioural profiles. Both clouds are then displayed together by projection onto planes, defined by factors. Each factor accounts for a certain proportion of the total variance of the cloud [28]. We here used grouped behaviours, grouped body postures, body orientations, distances to closest peer and locations as active variables (**Tables S1 and S2**). Hierarchical clustering analyses were then performed on the coordinates in the individuals’ cloud to describe inter-individual similarities [29]. This analysis sorts individuals on the dimensions defined by the previous MCA and creates clusters that maximize within-group similarity and minimize between-group similarity [29]. For each resulting cluster of individuals, the mean occurrence percentage of each behavioural item was calculated and reported on radar graphs.

### Statistical Analyses

The statistical analyse was conducted using Statistica^©^ 8.0 (StatSoft, Inc.). As data were not normally distributed, we used non-parametric statistical tests. Mann-Whitney U tests were used to compare variables between the two populations (captive- vs wild-born) [30]. Data from both populations were also separately submitted to Spearman order rank correlation tests to assess the impact of age and parturition number on the collected data.

When considering each population separately (captive- or wild-born), Kruskal-Wallis ANOVA followed by post-hoc Mann-Whitney U tests were used to compare variables between the clusters of individuals resulting from hierarchical analysis. Multiple tests were performed, a Bonferroni adjustment was thus applied to keep the type I error constant. The accepted P level becomes the α probability divided by the number of hypothesis tests: 0.003 (when 15 hypotheses). Considering the risk of masking significant effects following the correction, we chose to report also the cases of approximations to statistical significance (P<0.05). A correction for small group size was also applied when the group contained less than 10 individuals.

## Results

### Captive-born vs Wild-born

Behaviours, locations in the cage, body postures, orientations and distances to the closest peer were collected from 40 captive-born and 40 wild-born macaques using a scan sampling method. The mean behavioural time budgets of each population are reported in **Table 1**. Mann-Whitney U tests with origin as independent variable revealed statistical differences between captive- and wild-born animals for several items (**Table 1**). Although every monkey was adult, the age (wild: 6.7±0.2 years old and captive: 5.2±0.1 years old) and parturition number per female (wild: 2.0±0.0 infants and captive: 0.8±0.1 infants) were significantly higher in the wild-born population. However these two parameters were not significantly correlated in either population (**Table S3**). In the captive-born individuals, several behaviours, postures, orientations, locations and distances were significantly correlated with either age or the parturition number (**Table S3**). In contrast, in the wild-born animals age was correlated with only two behaviours and two locations in the cage, and the parturition number was correlated with none (**Table S3**).

**Table 1:** Occurrence of behaviours in 40 captive- and 40 wild-born monkeys and statistical comparisons.

Variables (mean % ± SEM)	Captive-born	Wild-born	MW U
age	5.2±0.1	6.7±0.2	287.0***
parturition number	0.8±0.1	2±0.0	231.0***
***Behaviours:***			
displacement B.:	1.2±0.4	1.7±0.2	528.5**
scratch	0.5±0.2	1.2±0.2	438.0***
vacuous chew	0.6±0.3	0.3±0.1	763.0
yawn	0.2±0.0	0.2±0.1	769.5
feeding B.	11.7±0.9	6.9±1.0	391.5***
B. toward human	0.2±0.1	0.3±0.1	693.5
inactivity:	42.5±2.2	51.8±1.8	492.0**
immobility	25.2±1.6	33.9±2.0	496.5**
resting B.	17.3±1.6	17.9±1.6	738.5
investigation	5.9±0.8	1.7±0.3	359.0***
locomotion	5.4±0.5	5.2±0.5	794.0
maternal B.	2.3±0.7	4.8±0.6	425.0***
maintenance B.	6.7±0.6	7.2±0.9	767.5
social B.:	17.0±0.9	18.7±1.0	690.5
agonistic B.	1.1±0.2	2.3±0.3	502.5**
stereotypic B.	6.5±1.3	1.2±0.3	373.0***
manual SB.	1.6±0.5	0.2±0.1	484.5***
motor SB.	2.0±0.9	0.2±0.2	467.0***
oral SB.	2.7±0.5	0.6±0.2	411.0***
**Behavioural diversity**	18.8±0.6	18.1±0.5	713.5
***Body postures:***			
seated	77.3±1.8	76.8±1.5	758.0
biped	0.6±0.1	0.3±0.1	582.5*
slumped	6.7±1.6	9.9±1.4	520.5**
lying down	0.9±0.2	2.2±0.4	647.0**
on bars	3.8±0.6	1.2±0.4	389.0***
four-legged:	9.6±1.1	9.0±0.6	731.5
crouched	1.0±0.2	0.5±0.1	591.0*
“bottom up”	1.2±0.2	0.8±0.1	549.0*
***Behaviours while slumped***	*n = 33*	*n = 39*	
displacement B.	0.1±0.1	0.2±0.1	632.0
inactivity	82.3±3.8	81.5±2.6	544.0
maternal B.	1.8±0.9	4.3±1.0	455.0**
maintenance B.	1.2±0.6	4.9±1.4	469.0*
social B.	13.4±3.6	8.6±1.6	619.0
***Body orientations:***			
peer	39.5±1.9	40.0±2.0	738.5
exterior	17.3±1.5	18.5±2	785.0
ground	10.7±1.1	10.2±0.6	707.0
wall	6.8±0.7	6.1±2.0	485.5**
open environment	25.6±2.4	25.2±2.7	783.5
***Behaviours while facing wall***	*n = 40*	*n = 39*	
feeding B.	12.9±2	25.6±3.8	559.5*
inactivity	27.1±2.9	37.6±3.7	579.0*
investigation	16.3±2.9	5.7±1.9	478.5**
maintenance B.	13.9±2	6.9±1.7	498.0**
social B.	14.4±2.7	11.6±2.0	716.0
stereotypic B.	9.0±1.9	1.8±0.7	510.5**
***Locations in the cage:***			
front	34.2±2.2	24.7±3.5	510.5**
back	34.6±2.0	40.8±3.7	661.0
bottom	31.5±2.5	17.9±3.1	354.0***
sitting bench	64.1±2.7	80.8±3.2	328.0***
up	4.4±0.8	1.3±0.4	397.5***
***Distances to nearest peer:***			
against	46.9±3.4	32.4±2.6	491.0**
d. <1arm	23.9±1.9	31.0±1.8	522.0**
1arm<d.<1 m	11.8±1.1	14.2±1.0	606.0
1 m<d.<3 m	16.4±1.6	20.2±1.7	620.5
d.>3 m	1.0±0.2	2.2±0.5	656.5

The mean percentages of occurrence (with regard to the 240 scans) and standard error means (SEM) per populations are reported below for a selection of collected variables. The behavioural diversity is the mean number of distinct behaviours observed during the 240 scans. The abbreviations “B.” and “d.” stand for behaviour and distance. Statistics and significant p-values in Mann-Whitney U tests comparing captive- and wild-born animals are reported in the right column (*: p<0.05;**: p<0.01;***: p<0.001).

Wild-born monkeys expressed more displacement behaviours (wild: 1.7±0.2% and captive: 1.2±0.4%), especially scratching (wild: 1.2±0.2% and captive: 0.5±0.2%), more immobility (wild: 33.9±2.0% and captive: 25.2±1.6%), more maternal behaviours (wild: 4.8±0.6% and captive: 2.3±0.7%), and more agonistic behaviours (wild: 2.3±0.3% and captive: 1.1±0.2%) than captive-born, while captive-born individuals expressed more feeding (wild: 6.9±1.0% and captive: 11.7±0.9%), more investigation (wild: 1.7±0.3% and captive: 5.9±0.8%), and more stereotypic behaviours regardless of the category (wild: 1.2±0.3% and captive: 6.5±1.3%). Moreover play behaviours, included in the “investigation” category, were expressed at least once by 25% of captive- (two of these animals were 4 years old, six were 5 years old and two were 6 years old) and 2.5% of wild-born individuals (6 years old) (data not shown). SB were expressed at least once by 82.5% of the captive- compared to 60.0% of the wild-born population (data not shown). Wild animals were more often in a slumped body posture (wild: 9.9±1.4% and captive: 6.7±1.6%) and lying down while captive ones stood on their feet (wild: 0.3±0.1% and captive: 0.6±0.1%), were on bars (wild: 1.2±0.4% and captive: 3.8±0.6%), crouched (wild: 0.5±0.1% and captive: 1.0±0.2%) or displayed the “bottom up” posture (wild: 0.8±0.1% and captive: 1.2±0.2%). Captive animals were more often facing the wall (wild: 6.1±2.0% and captive: 6.8±0.7%), but this body orientation was associated with investigation (wild: 5.7±1.9% and captive: 16.3±2.9%), maintenance (wild: 6.9±1.7% and captive: 13.9±2.0%) or SB (wild: 1.8±0.7% and captive: 9.0±1.9%), while associated with inactivity (wild: 37.6±3.7% and captive: 27.1±2.9%) or feeding (wild: 25.6±3.8% and captive: 12.9±2.0%) in wild monkeys. Wild animals were located mostly on the sitting benches (wild: 80.8±3.2% and captive: 64.1±2.7%) compared to captive monkeys which were often seen at the bottom (wild: 17.9±3.1% and captive: 31.5±2.5%) or in the upper (wild: 1.3±0.4% and captive: 4.4±0.8%) parts of the cage and approached the front (wild: 24.7±3.5% and captive: 34.2±2.2%) part more frequently. Finally, while wild individuals stayed within 1 arm of their peers (wild: 31.0±1.8% and captive: 23.9±1.9%), captive ones were often against them (wild: 32.4±2.6% and captive: 46.9±3.4%).

### Captive-born Animals

**Figure 1:**
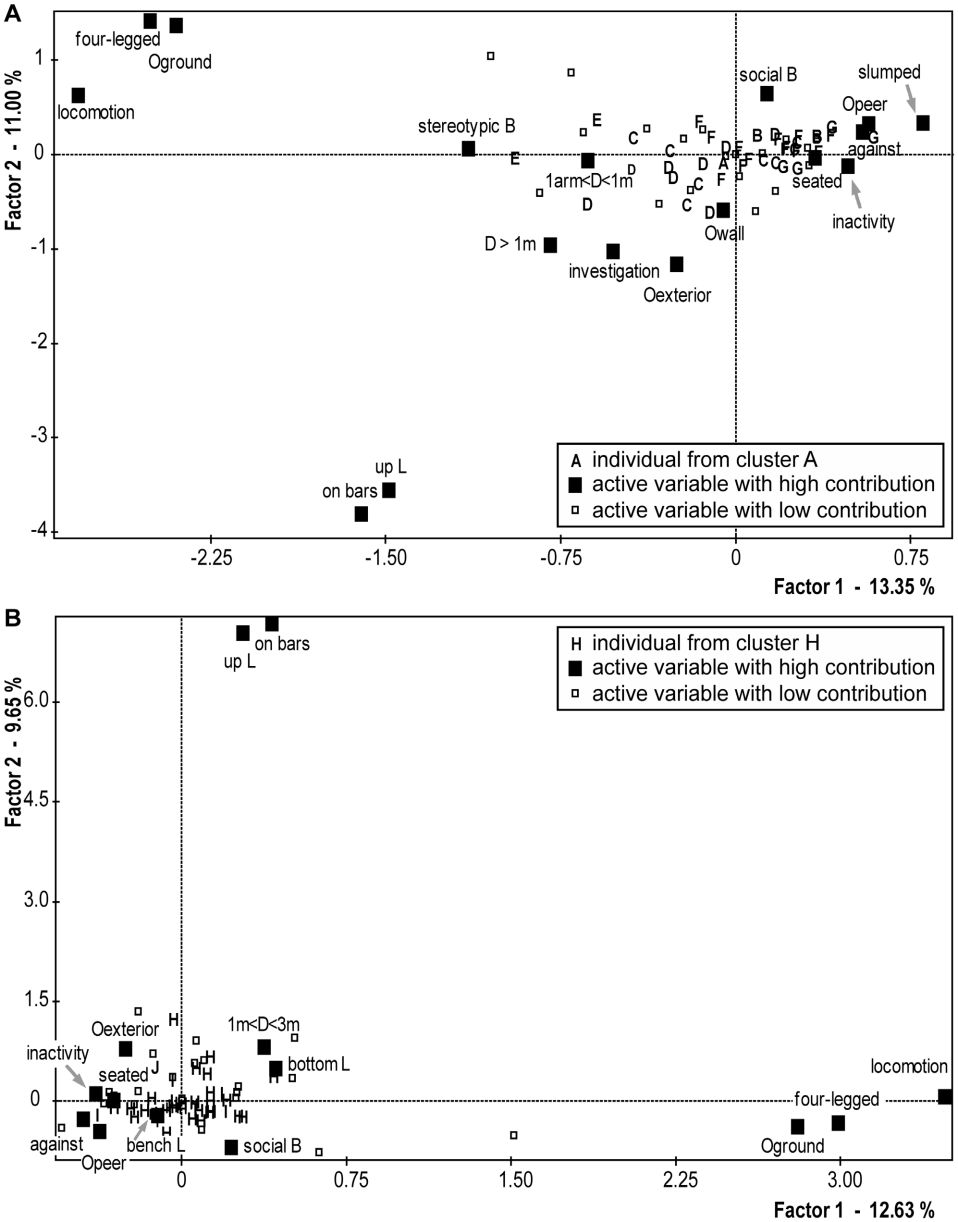
First factorial plane of the multiple component analyses (MCA) in captive-and wild-born animals. Behaviours, postures, distances to nearest peer, body orientations and locations expressed by the 40 socially-housed captive- (panel A) or wild- (panel B) born cynomolgus monkeys were submitted to MCA. The individuals are represented by bold black letters, accounting for the clusters to which they belong according to the cluster analysis following the MCA. Squares represent active modalities: grouped behaviours, grouped postures, distances to nearest peer, body orientations and locations in the cage. On each axis is reported the percentage of the total variance accounted for by each factor. The abbreviations “B”, “D”, “L” and “O” stand for “behaviour”, “distance”, “location” and “orientation”. See **Table S1 and S2** for a detailed description of each variable.

A multiple component analysis (MCA) was performed in order to analyse the great inter-individual variability among the considerable number of assessed behavioural items (listed in **table S1** and **S2**) in captive-born monkeys (**Figure 1** **, panel A)**. The first factorial plane, defined by two dimensions (*i.e.* factor 1 and factor 2) of the analysis accounted for 24.3% of the total variance (factor 1 accounted for 13.3% and factor 2 for 11.0%). As explained in the Material and Methods section, this analysis showed which combinations of collected variables were often expressed together and how important these combinations are to explain the inter-individual variability. The further from 0 a variable is, the more this variable contributes to the variance of the sample. On the first axis, the locomotion, stereotypic behaviours (SB), four-legged posture (and therefore the “toward ground” body direction), “on bars” posture and the distance “more than 1 m” were strongly opposed to inactivity in a seated posture and body directed toward- and located against- a peer. On the second axis, the upper location on bars, the body orientation toward the exterior of the cage, investigation behaviours and the distance “more than 1 m” were opposed to social behaviours in a four-legged posture (and therefore facing the ground).

**Figure 2:**
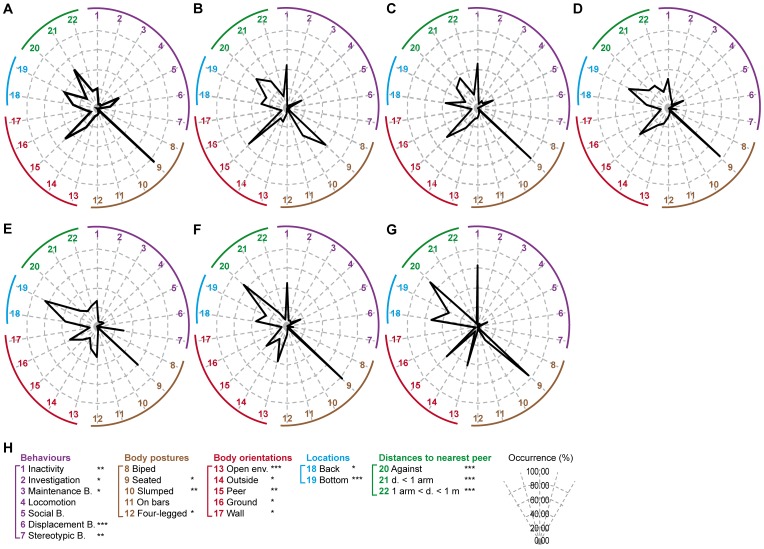
Seven behavioural profiles resulting from hierarchical cluster analysis in captive-born animals. Following the MCA of the captive-born animals, a hierarchical cluster analysis was performed and resulted in 7 groups (n_A_ = 1, n_B_ = 3, n_C_ = 8, n_D_ = 8, n_E_ = 2, n_F_ = 14, n_G_ = 4). For each variable collected, the mean percentages of occurrence were calculated among the 7 groups. The radar profiles of group A (**panel** **A)**, group B (**panel B**), group C (**panel** **C)**, group D (**panel D**), group E (**panel** **E)**, group F (**panel** **F**) and group G (**panel** **G)** were created using a selection of collected variables (**panel** **H**). The radar legend of the radars is explained on panel **H**. Each axis of the radar indicates the mean percentage of occurrence for a given variable: a behaviour (purple numbers from 1 to 7), a body posture (brown numbers from 8 to 12), a body orientation (red numbers from 13 to 17), a location in the cage (blue numbers from 18 to 19) or a distance to nearest peer (green numbers from 20 to 22). The abbreviations “B.”, “d.” and “env.” stand for “behaviour”, “distance” and “environment”. Significant p-values after Kruskal-Wallis tests are indicated by stars (*: p<0.05;**: p<0.01;***: p<0.001).

Data were then submitted to hierarchical cluster analysis in order to accurately identify the groups of individuals displaying similar profiles. This resulted in seven distinct clusters, named A (n = 1), B (n = 3), C (n = 8), D (n = 8), E (n = 2), F (n = 14) and G (n = 4) groups. Salient results are displayed on radar graphs in **Figure 2** while comprehensive statistical analysis is presented in **Table 2**.

**Table 2:** Occurrence of behaviours in 7 captive-born clusters and statistical comparisons.

H_(6,40)_	KW p	Variables (M% ± SEM; MW p)	A; n = 1	group B; n = 3		group C; n = 8		group D; n = 8		group E; n = 2		group F; n = 14		group G; n = 4	
5.22	0.503	**mean age**	4	5.0±0.6		5.5±0.3		4.9±0.2		5.0±1.0		5.5±0.2		5.0±0.6	
8.87	0.181	**mean parturition number**	0	1.7±0.3		0.7±0.2		0.4±0.3		0.5±0.5		1.1±0.2		0.5±0.3	
		***Behaviours:***													
22.41	**0.001**	displacement B.:	14.7	3.2±1.1	cF"g"	0.7±0.3	bd	1.8±0.3	ceFg	0.2±0.2	d	0.3±0.1	BD	0.2±0.2	"b"d
21.78	**0.001**	scratch	0.0	2.8±0.9	cF"g"	0.1±0.1	b	1.1±0.5	"f"	0.2±0.2	"f"	0.0±0.0	B"d""e"	0.0±0.0	"b"
15.55	**0.016**	vacuous chew	13.9	0.1±0.1		0.3±0.2		0.6±0.2	fg	0.0±0.0		0.1±0.1	d	0.0±0.0	d
9.65	0.140	feeding B.	22.3	10.5±0.6		9.4±1.9		14.5±2.4		17.1±2.8		9.8±1.5		13.2±1.6	
17.64	**0.007**	B. toward human	0.0	0.6±0.1	df"g"	0.6±0.4		0.0±0.0	b	0.0±0.0		0.0±0.0	b	0.1±0.1	"b"
20.47	**0.002**	inactivity:	*21.0*	43.9±6.6	"g"	46.3±3.4	deg	30.6±3.6	cfg	26.4±5.8	c	44.4±2.7	dG	64.3±3.9	"b"cdF
24.99	**0.000**	Immobility	12.2	12.9±2.3	cf"g"	22.2±1.6	bFg	17.8±2.9	Fg	17.3±3.0	f	32.8±2.0	bCDe	36.1±4.0	"b"cd
22.02	**0.001**	resting B.	8.8	31.1±8.8		24.0±3.2	deF	12.8±3.1	cg	9.1±2.8	c	11.6±1.1	CG	28.2±3.4	dF
14.44	**0.025**	investigation	4.6	2.1±0.6	d"g"	4.5±1.1	dg	11.2±2.3	bcg	5.7±2.3		6.1±1.3		0.9±0.2	"b"cd
8.50	0.204	locomotion	4.6	4.0±1.5		7.4±1.9		5.3±1.1		7.0±2.3		5.4±0.6		2.2±0.7	
15.99	**0.014**	maternal B.	0.0	12.0±2.3	cdf"g"	2.0±1.1	b	0.0±0.0	b"f"	0.0±0.0		2.8±1.3	b"d"	0.4±0.4	"b"
12.95	**0.044**	maintenance B.	5.5	*1.8±0.3*	cdf"g"	8.8±1.9	bg	8.3±1.4	bg	5.3±1.0		6.7±0.7	b	4.1±0.5	"b"cd
11.35	0.078	social B.	26.9	18.2±3.1		19.1±1.5		18.6±1.8		*8.2±0.7*		16.4±1.2		12.1±4.2	
18.58	**0.005**	stereotypic B.	0.4	3.0±0.5		0.9±0.5	Def	9.6±2.5	Ce	30.1±3.4	cdf	7.1±1.6	ce	2.9±1.0	
15.91	**0.014**	**Behavioural diversity**	*14.0*	*23.0±2.5*	*c"g"*	*16.1±1.0*	*bdf*	*20.7±1.2*	*cg*	*20.5±4.5*		*19.6±0.6*	*cg*	*15.0±0.8*	*"b"df*
		***Body postures:***													
14.12	**0.028**	seated	85.3	58.0±6.4	cdF"g"	79.8±4.7	b	76.5±3.3	be	*60.5±3.8*	df	82.5±1.5	Be	76.6±2.3	"b"
6.14	0.408	biped	0.0	0.4±0.0		0.4±0.1		0.9±0.4		0.2±0.2		0.8±0.2		0.2±0.2	
20.43	**0.002**	slumped	1.7	33.7±7.5	cdF	2.6±2.0	bd"f"g	3.2±0.8	bcg	1.1±1.1		3.8±0.9	B"c"G	15.8±3.8	cdF
9.03	0.172	on bars	5.0	1.1±0.6		5.9±2.0		6.3±1.6		4.8±4.8		2.0±0.5		1.9±0.9	
13.25	**0.039**	four-legged	6.7	6.0±1.4		10.1±2.5	eg	9.9±1.5	eg	31.7±0.2	cdf	8.6±1.1	eg	4.1±0.9	cdf
**H_(6,33)_**		***Main B. while slumped:***				***n = 3***				***n = 1***		***n = 13***			
9.02 ^(6,33)^	0.172	inactivity	100.0	59.7±9.4		73.6±16.1		84.4±7.0		80.0		81.7±8.2		99.6±0.4	
25.02 ^(6,33)^	**0.000**	maternal B.	0.0	17.5±4.1	"c"df"g"	0.0±0.0		0.0±0.0	b	0.0		0.5±0.5	b	0.0±0.0	"b"
5.72 ^(6,33)^	0.455	social B.	0.0	13.6±4.3		21.1±17.3		11.5±6.3		20.0		17.4±8.4		0.4±0.4	
		***Body orientations:***													
22.15	**0.001**	peer	48.1	56.2±3.2	F	46.1±3.0	eF	41.4±3.8	ef	*20.9±4.8*	cd	31.1±1.9	BCdg	46.5±5.6	f
14.21	**0.027**	exterior	23.2	12.7±1.0	c"g"	24.4±1.8	bfg	21.1±2.9	g	14.1±10.3		15.6±2.7	cg	*5.0±1.0*	"b"cdf
14.67	**0.023**	ground	8.0	6.2±1.3		10.7±2.4	e	12.6±1.8	eg	33.0±0.1	cdf	9.3±1.1	eg	4.9±1.1	df
13.65	**0.034**	wall	12.7	10.1±2.1	"g"	6.8±1.7	g	8.1±1.1	g	8.0±2.5		6.2±1.1	g	2.1±0.4	"b"cdf
26.03	**0.000**	open environment	8.0	14.8±1.4	F"g"	11.9±1.7	Fg	16.7±3.6	Fg	23.9±12.5		37.9±2.7	BCD	41.5±5.8	"b"cd
		***Locations in the cage:***													
5.26	0.511	front	39.0	42.5±12.7		32.3±6.5		38.4±6.1		33.3±5.3		34.6±2.5		*21.0±3.1*	
13.53	**0.035**	back	27.5	29.0±6.9	"g"	36.8±4.6	g	*24.3±4.1*	fg	35.5±2.2		35.5±2.8	dg	53.1±2.8	"b"cdf
25.88	**0.000**	bottom	40.3	25.2±2.0	d	*15.1±2.5*	Defg	48.0±4.6	bCF	63.4±0.3	cf	26.2±2.6	cDe	35.9±4.8	c
25.55	**0.000**	sitting bench	55.5	73.9±2.0	d	79.1±2.5	Deg	43.5±3.2	bCF	*33.9±2.9*	cf	71.4±2.8	De	59.6±7.2	c
6.37	0.383	up	4.2	0.8±0.5		5.8±1.8		8.4±2.8		2.6±2.6		2. ±0.6		4.5±2.5	
		***Distances to nearest peer:***													
29.06	**0.000**	against	*15.7*	45.3±4.3	df"g"	31.6±4.0	Fg	28.4±4.2	bFg	24.7±14.0	f	64.9±2.9	bCDe	71.3±6.7	"b"cd
29.48	**0.000**	d. <1arm	48.5	32.3±2.4	F"g"	37.0±3.0	eFg	27.7±3.8	fg	18.8±2.7	c	16.0±0.8	BCdG	*7.6±0.8*	"b"cdF
27.15	**0.000**	1arm<d.<1 m	19.1	12.4±2.3	"g"	14.5±1.5	Fg	17.7±2.0	Fg	21.9±4.5	f	7.0±1.0	CDe	*3.6±0.9*	"b"cd
14.42	**0.025**	1 m<d.<3 m	15.7	9.6±0.5	d	15.4±2.4	e	24.3±3.6	bf	34.2±6.6	cf	11.7±2.1	de	15.9±4.7	
13.65	**0.034**	d.>3 m	0.9	0.3±0.3		1.5±0.4	f	1.8±0.5	f	0.2±0.2		0.4±0.2	cd	1.4±0.8	

The mean percentages (M) of occurrence (with regard to the 240 scans) and standard error means (SEM) per cluster are reported below for a selection of collected variables. The behavioural diversity is the mean number of distinct behaviours observed during the 240 scans. The abbreviations “B.” and “d.” stand for behaviour and distance. Kruskal-Wallis statistics and p-values are indicated in the first two left columns, bold font highlighting significance. Significant p-values in post-hoc Mann-Whitney U tests before (small-letters, p<0.05) and after (capital letters, p<0.005) a Bonferroni adjustment are indicated in columns at the right of the occurrence percentages. The letters represent the groups versus which the p-values are significantly different for a given variable. P-values between quotation marks (« ») indicate significance (p<0.05) if small group size correction was not applied.

In the “main behaviours while slumped” section, the number of monkeys expressing the slumped body posture is specified for clusters C, E and F as all monkeys did not display this posture (n = 33 displayed the slumped posture). Here the mean percentages of occurrence with regard to the number of scans spent in the slumped body posture are reported for each cluster.

The 7 groups presented a few similarities such as the levels of locomotion (A: 4.6%; B: 4.0±1.5%, C: 7.4±1.9%, D: 5.3±1.1%, E: 6.9±2.3%, F: 5.4±0.6%, and G: 2.2±0.7%) and social behaviours (A: 26.9%; B: 18.2±3.1%, C: 19.1±1.5%, D: 18.6±1.8%, E: 8.2±0.7%, F: 16.4±1.2%, and G: 12.1±4.2%), the majority of time spent seated (A: 85.3%; B: 56.0±6.4%, C: 80.0±4.8%, D: 76.5±3.3%, E: 60.5±3.7%, F: 82.5±1.5%, and G: 76.6±2.3%) on the sitting benches (A: 55.5±1.2%; B: 73.9±2.0%, C: 79.1±2.5%, D: 43.5±3.2%, E: 33.9±2.9%, F: 71.4±2.8%, and G: 59.6±7.2%), except for groups D and E, the low occurrences of the “on bars” body posture (A: 5.0%; B: 1.1±0.6%, C: 5.9±2.0%, D: 6.3±1.6%, E: 4.8±4.8%, F: 2.0±0.5%, and G: 1.9±0.9%), and the most frequent orientation of the body toward peers, except for groups E and F (A: 48.1%; B: 56.2±3.2%, C: 46.1±3.0%, D: 41.4±3.8%, E: 20.9±4.8%, F: 31.1±1.9%, and G: 46.5±5.6%). Nevertheless, many differences explained the partition of the population in 7 clusters (**Figure 2** **;** **Table 2**).

A Group (**Figure 2A** **;** **Table 2**) included only one individual and therefore could not be submitted to the Mann-Whitney U tests. It was however characterized by a high level of displacement (14.7%; especially vacuous chewing: 13.9%), feeding (22.3%) and social (26.9% divided in 23.1% grooming peers, 3.4% being groomed and 0.4% submissive behaviours; data not shown) behaviours, the lowest amount of inactivity (21.0%) and lowest behavioural diversity (14 behaviours) compared to others groups. It spent much time facing the exterior of the cage (23.2%) or the wall (12.6%). This monkey was located 40.2% of the scans at the bottom of the cage and 48.5% at less than 1 arm from its peers and very few against peers compared to others groups.

B Group (**Figure 2B** **;** **Table 2**) displayed a few displacement activities (3.2±1.1%, especially scratching: 2.8±0.9%), a low level of maintenance behaviours (1.8±0.3%) and the maximum behavioural diversity (23.0±2.5 behaviours). The animals expressed the highest level of maternal behaviours (A: 0.0%; B: 12.0±2.3%, C: 2.0±1.1%, D: 0.0±0.0%, E: 0.0±0.0%, F: 2.8±1.3%, and G: 4.1±0.5%) although this result must be taken cautiously because not every female had an infant during the study. They spent the lowest amount of time seated (58.0±6.4%) and the highest number of scans in a slumped posture (33.7±7.5%). When slumped, they spent 59.7% (±9.4%) of the time inactive, 17.5% (±4.1%) expressing maternal behaviours and 13.6% (±4.3%) in social behaviours. This group oriented its body toward peers the most (56.2±3.2%). The percentage of time spent at the front of the cage was not significantly different from the other groups, though twice the level of G group (B: 42.5±12.7% and G: 21.0±3.1%).

Individuals from C group (**Figure 2C** **;** **Table 2**) expressed intermediate level of each behaviour, except for maintenance activities that were highly expressed (8.8±1.9%), but they displayed the lowest behavioural diversity (16.1±1.0 behaviours) after the A individual. They rarely expressed the slumped body posture (2.6±2.0%) and their body was often oriented toward the exterior of the cage (24.4±1.8%). They spent least of the time at the bottom of the cage (15.1±2.5%) and at less than 1 arm from their peer (37.0±3.0%).

D Group (**Figure 2D** **;** **Table 2**) expressed a few displacement behaviours (1.8±0.3%), mostly scratching (1.1±0.5%), a low level of inactivity (30.6±3.6%), the highest level of cage investigation (11.2±2.3%), a high amount of maintenance activities (8.3±1.4%) and many SB (9.6±2.5%, especially oral SB: 6.0±1.8%). These animals spent little time at the back of the cage (24.3±4.1%) and much time at the bottom or in the upper parts of the cage (bottom: 48.0±4.6%; up: 8.4±2.8%).

The main characteristics of E group (**Figure 2E** **;** **Table 2**) were a low level of inactivity (26.4±5.8%, mainly in resting) and social behaviours (8.2±0.7%), and the highest level of SB (30.1±3.4%, especially motor SB: 24.3±0.3%) and four-legged body posture (31.7±0.2%), therefore associated with a frequent orientation toward the ground (33.0±0.1%). Their bodies were the least often oriented toward peers (20.9±4.8%) and the most often located at the bottom of the cage (63.4±0.3%). They stood mainly between 1 arm and 3 m from their peers (1arm<d.<1 m: 21.9±4.5%; 1 m<d.<3 m: 34.2±6.6%).

Individuals from F group (**Figure 2F** **;** **Table 2**) presented no major characteristics as they expressed intermediate levels of each item. They expressed a few SB (7.1±1.6%) and a high behavioural diversity (19.6±0.6 behaviours). They faced the open environment more often than most other groups did (A: 8.0%; B: 14.8±1.4%, C: 11.9±1.7%, D: 16.7±3.6%, E: 23.9±12.5%, F: 37.9±2.7%, and G: 41.5±5.8%) and they faced peers less often than most groups (A: 48.1%; B: 56.2±3.2%, C: 46.1±3.0%, D: 41.4±3.8%, E: 20.9±4.8%, F: 31.1±1.9%, and G: 46.5±5.6%). They stood most of the time against a peer (64.9±2.9%).

Finally, G group (**Figure 2G** **;** **Table 2**) spent most of the time inactive (64.3±3.9%) and therefore displayed a low behavioural diversity (15.0±0.8 behaviours). They seated in a slumped posture 15.8% (±3.8%) of the scans, showed the highest levels of location at the back of the cage (53.1±2.8%), body orientation toward the open environment (41.5±5.8%) and closeness to peers (against: 71.3±6.7%). When slumped, they stayed inactive in 99.6% (±0.4%) of the scans.

### Wild-born Animals

A MCA was also performed on the assessed behavioural items in wild-born monkeys (**Figure 1** **, panel B)**. The first factorial plane of this analysis accounted for 22.3% of the total variance (factor 1 accounted for 12.6% and factor 2 for 9.7%). Although the first and second axes were graphically different from the ones in the captive MCA, the same variable oppositions in the captive-born individuals characterize them.

**Figure 3:**
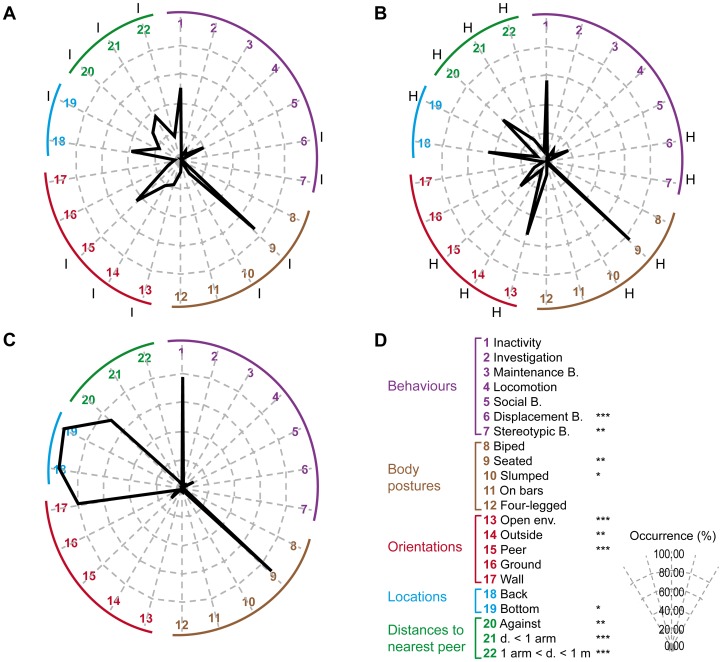
Three behavioural profiles resulting from hierarchical cluster analysis in wild-born animals. Following the MCA of the wild-born animals, a hierarchical cluster analysis was performed and resulted in 3 groups (n_H_ = 31, n_I_ = 8, n_J_ = 1). For each variable collected, the mean percentages of occurrence were calculated among the 3 groups. The radar profiles of group H (**panel** **A)**, group I (**panel** **B)**, and group J (**panel** **C)** were created using a selection of collected variables (**panel** **D**). The radar legend of the radars is explained on panel **D**. Each axis of the radar indicates the mean percentage of occurrence for a given variable: a behaviour (purple numbers from 1 to 7), a body posture (brown numbers from 8 to 12), a body orientation (red numbers from 13 to 17), a location in the cage (blue numbers from 18 to 19) or a distance to nearest peer (green numbers from 20 to 22). The abbreviations “B.”, “d.” and “env.” stand for “behaviour”, “distance” and “environment”. Significant p-values after Kruskal-Wallis tests are indicated by stars (*: p<0.05;**: p<0.01;***: p<0.001). On graphs **A and B**, significant p-values in Mann-Whitney U tests are indicated (p<0.05) by capital letters (H or I: versus cluster H or I respectively). As cluster J (**panel C**) contains only 1 animal, Mann-Whitney U tests could not be performed with its data.

The hierarchical cluster analysis of the wild-born animals resulted however in only three distinct clusters, named H (n = 31), I (n = 8), and J (n = 1) (**Figure 3**). Salient results are displayed on radar graphs in **Figure 3** while comprehensive statistical analysis is presented in **Table 3**.

**Table 3:** Occurrence of behaviours in 3 wild-born clusters and statistical comparisons.

H_(2,40)_	KW p	Variables (mean % ± SEM)	group H	group I	MW U	J (%)
		**group size**	31	8		1
2.21	0.331	**mean age**	6.7±0.2	6.7±0.5	123.0	5
39.00	**0.000**	**mean parturition number**	2.0±0.0	2.0±0.0	124.0	1
		***Behaviours:***				
14.64	**0.001**	displacement B.:	2.1±0.2	0.2±0.2	20.0***	0.4
18.26	**0.000**	scratch	1.5±0.2	0.0±0.0	8.0***	0.0
10.98	**0.004**	feeding B.	8.2±1.2	1.7±0.7	30.0***	6.7
5.19	0.075	B. toward human	0.3±0.1	0.0±0.0	72.0	0.0
5.73	0.057	inactivity	49.6±2.0	56.6±2.9	74.0	77.7
14.59	**0.001**	immobility	29.8±1.6	45.7±3.8	23.0***	66.0
7.49	**0.024**	resting B.	19.8±1.8	10.9±2.4	50.0**	11.8
0.88	0.644	investigation	1.7±0.3	1.8±0.7	113.5	2.1
28.75	**0.000**	object handling	0.0±0.0	1.7±0.7	19.0***	1.7
3.21	0.201	locomotion	5.1±0.4	6.1±1.4	106.5	*0.4*
3.25	0.197	maternal B.	4.7±0.8	5.5±0.9	94.0	0.0
8.19	**0.017**	negative maternal B.	0.1±0.1	0.7±0.3	56.0*	0.0
3.28	0.194	maintenance B.	6.8±0.9	9.3±2.2	89.0	2.5
2.61	0.270	social B.	19.1±1.2	18.1±2.1	117.5	*9.2*
11.44	**0.003**	stereotypic B.	1.5±0.4	0.0±0.0	32.0***	0.8
12.10	**0.002**	**Behavioural diversity**	19.0±0.5	15.0±0.9	33.0***	*13.0*
		***Body postures:***				
9.85	**0.007**	seated	74.6±1.7	83.9±1.9	46.0**	89.9
0.55	0.760	biped	0.3±0.1	0.4±0.3	119.0	0.0
8.16	**0.017**	slumped	11.8±1.6	3.3±0.9	44.5**	5.5
2.57	0.276	on bars	1.5±0.5	0.4±0.2	90.5	0.0
2.69	0.261	four-legged	9.1±0.7	9.4±1.6	113.0	3.4
		***Main B. while slumped:***	**n = 30**			
16.04	**0.000**	inactivity	76.4±2.8	99.0±1.1	10.5***	92.3
7.92	**0.019**	maternal B.	5.5±1.3	0.0±0.0	52.0*	0.0
12.69	**0.002**	social B.	10.9±1.9	0.0±0.0	24.0***	7.7
		***Body orientations:***				
16.03	**0.000**	peer	44.4±1.8	26.4±2.7	17.0***	11.3
13.26	**0.001**	exterior	22.1±1.9	6.8±4.1	28.0***	0.4
2.35	0.309	ground	10.3±0.7	10.4±1.6	116.5	4.6
5.24	0.073	wall	4.5±0.7	2.8±1.0	78.5	80.7
20.55	**0.000**	open environment	18.6±1.5	53.6±3.6	0.0***	2.9
		***Main B. while facing wall:***		**n = 7**		
1.78	0.410	feeding B.	26.7±3.7	23.4±14.4	80.0	7.3
6.91	**0.032**	immobility	27.3±3.3	44.1±8.8	54.0*	74.0
4.42	0.110	social B.	13.4±2.2	5.2±5.2	57.0	2.1
		***Locations in the cage:***				
4.15	0.125	front	27.1±3.7	18.4±9.0	80.0	1.7
3.27	0.195	back	37.9±3.6	45.1±10.6	105.0	95.4
9.155	**0.010**	bottom	17.9±2.7	7.8±4.0	50.0**	99.6
8.82	**0.012**	sitting bench	80.6±2.9	91.6±4.1	52.0*	0.4
1.16	0.559	up	1.5±0.5	0.6±0.3	116.0	0.0
		***Distances to nearest peer:***				
10.22	**0.006**	against	28.0±2.6	44.2±3.5	44.0**	72.2
17.29	**0.000**	d. <1arm	35.2±1.6	17.9±1.6	12.0***	5.3
17.55	**0.000**	1arm<d.<1 m	16.5±0.9	6.9±0.7	11.0***	1.8
1.65	0.439	1 m<d.<3 m	19.4±1.9	24.4±3.8	96.0	10.6
16.96	**0.000**	d.>3 m	0.8±0.2	6.5±1.3	13.5***	10.1

The mean percentages of occurrence (with regard to the 240 scans) and standard error means (SEM) per cluster are reported below for a selection of collected variables. The behavioural diversity is the mean number of distinct behaviours observed during the 240 scans. The abbreviations “B.” and “d.” stand for behaviour and distance. Kruskal-Wallis statistics and p-values are indicated in the first two left columns, bold font highlighting significance. As cluster J contained only 1 animal, post-hoc Mann-Whitney U tests could only be performed to compare groups H and I. These statistics are reported in the “MW U” column and associated significant p-values are indicated by stars (*: p<0.05;**: p<0.01;***: p<0.001).

H and I groups (**Figure 3A–B**, respectively, **Table 3**) showed several similarities in behavioural occurrences such as investigation (H: 1.7±0.3% and I: 1.8±0.7%), locomotion (H: 5.1±0.4% and I: 6.1±1.4%), maintenance (H: 6.8±0.9% and I: 9.3±2.2%) and social behaviours (H: 19.1±1.2% and I: 18.1±2.1%). However individuals from H cluster expressed significantly more displacement (H: 2.1±0.2% and I: 0.2±0.2%), feeding (H: 8.2±1.2% and I: 1.7±0.7%), resting (H: 19.8±1.8% and I: 10.9±2.4%) and stereotypic (H: 1.5±0.4% and I: 0.0±0.0%) behaviours than I group, while I cluster displayed more immobility (H: 29.8±1.6% and I: 45.7±3.8%) than H, though no more significantly different when pooling immobility and resting behaviours (inactivity: H 49.6±2.0% and I 56.6±2.9%). Similarly, the behavioural diversity was significantly higher in H group (19.0±0.5 behaviours vs 15.0±0.9 in I group). Both groups spent most of their time sitting and displayed the same level of four-legged posture (H: 9.1±0.7% and I: 9.4±1.6%) but the seated proportion was maximal in I group (H: 74.6±1.7% and I: 83.9±1.9%) while H group showed a high level of slumped body posture (H: 11.8±1.6% and I: 3.3±0.9%). The main differences appeared in terms of body orientations, locations in the cage and distances to nearest peer (**Table 3**). While H group was mainly oriented towards peer (H: 44.4±1.8% and I: 26.4±2.7%) and the exterior of the cage (H: 22.1±1.9% and I: 6.8±4.1%), I group was oriented toward the open environment (H: 18.6±1.5% and I: 53.6±3.6%). Both groups spent most of their time on sitting benches (H: 80.6±2.9% and I: 91.6±4.1%) but H animals were at the bottom of the cage more often than I animals (H: 17.9±2.7% and I: 7.8±4.0%). The most frequent distance separating animals was one arm for H group (H: 35.2±1.6% and I: 17.9±1.6%) and none for I group (H: 28.0±2.6% and I: 44.2±3.5%).

The 3^rd^ cluster (**Figure 3C** **,** **Table 3**) resulting from the partition of the hierarchical cluster analysis contained only one individual, thereby preventing us from testing the statistical differences with the previous groups. Nevertheless this animal clearly presented a distinct behavioural profile with a high level of inactivity (77.7%, mainly immobility: 66.0%), and low levels of locomotion (0.4%), maintenance (2.5%) and social (9.2%) behaviours. The number of different behaviours expressed during the 240 scans was quite low (13.0 behaviours). J monkey also spent most of its time facing the wall (80.7%), at the back bottom (back: 95.4%; bottom: 99.6%) of the cage against a peer (72.2%).

## Discussion

We here described the daily life behavioural profiles of eighty socially-housed adult female cynomolgus monkeys living in a farming environment. We investigated the differences likely related to distinct early life origins on these profiles by comparing captive- and wild-born individuals and highlighted a few general significant differences between these two populations living in the same environment, such as the higher proportions of stereotypic behaviours (SB) and feeding among the captive-born individuals opposed to a higher level of inactivity among the wild-born animals. Using multifactorial analyses and hierarchical clustering, we then identified 7 and 3 distinct behavioural profiles in the captive- and wild-born populations respectively, which differed significantly from one another on several behaviours as well as on their body postures, body orientations and location in the cage. One captive-born (G group) and one wild-born (J individual) profiles seemed reminiscent of a depressive symptomatology. The other profiles did not clearly mimic any other known mental disorder.

### Captive vs Wild

Captive-born individuals expressed significantly more investigation, feeding and stereotypic behaviours (SB) whereas wild-born animals expressed more inactivity, maternal, agonistic and displacement behaviours. Several factors, such as age, number of parturition, hierarchical rank, temperament, or early life experience, have been shown to affect inter individual behavioural differences.

Age was indeed significantly lower in the captive-born population, though all individuals were adult. Some behaviours (*e.g.* play) are known to decreased with age [31], [32]. Investigation (that included play, environment investigation and object manipulation in our study) was indeed inversely correlated with age in the wild-born animals. However this correlation did not occur in the captive-born population nor if play behaviours were tested alone. Other behavioural changes (*e.g.* increase of rest, decrease of allogrooming and aggressions received) due to age [33] were not observed in our study. Play has also been suggested as a stress reducer in marmosets where dominants scratched a lot and rarely played while subordinates often played and rarely scratched [34] or in chimpanzees where play increased during tensed pre-feeding periods [35]. In other species, such as horses, the frequency of adult play has been reported as correlated with a global score of chronic stress [36]. Could play and investigation behaviours be a buffering tool in tension reduction in adult captive-born animals whereas wild-born individuals resort to classic displacement behaviours?

Displacement behaviours, especially scratching, were indeed significantly higher in the wild-born population. Although it has been shown that birth season and infant presence were associated with enhanced emotionality (*i.e.* higher scratching rate) in rhesus macaques [37], the number of parturition was not correlated to these behaviours in either population. The number of parturition was not correlated with any variables in the wild-born animals while, in the captive-born monkeys, it was positively associated with agonistic behaviours, manual SB and behavioural diversity and negatively correlated with feeding, maintenance and long inter-individual distances. Wild cynomolgus monkeys follow a well-established hierarchical system with dominants having priority access to food, groomers and best resting spots, and subordinates being punished (threatened, chased or bitten by dominants) whenever they do not respect these social “rules” [31], [38], [39]. With significantly higher feeding level and fewer agonistic encounters, could it be that captive-born individuals display a more permissive (*i.e.* atypical) hierarchy? The breeding processes employ an early weaning and a same age peer-rearing from 6 months old to 3 years old, thereby disrupting the mother-to-daughter rank heritability which is maintained by social rather than genetic transmission [40]. Moreover, the absence of adults could prevent observational learning and imitation of appropriate behaviours and responses in social encounters. A recent study on farm horses compared peer-weaned foals with foals weaned with 2 unrelated adults and reported that the presence of these adults lowered the expression of abnormal behaviours, increased social cohesion and normalized the time budgets of the foals [41]. In NHPs, though production of communication signals seems rather innate, the appropriate interpretation of these signals might be influenced by social environment [40], [42]. For instance, after aggressive encounters rhesus macaques, reared with stumptail macaques, expressed reconciliation, which is commonly expressed in the second species but rarely among rhesus [43]. Given the wealth of studies reporting long and short term physiological and behavioural consequences following suboptimal rearing conditions [2], [3], [10], [11], [44]–[46], the disrupted early life experiences seem the most likely explanation for the behavioural differences observed between our captive- and wild-born subjects. The higher expression of SB and higher proportion of individuals expressing SB in the captive-born population further support this idea since very few SB were reported in the wild compared to captive environments with or without social “wild-like” enclosures [9], [19], [47], [48]. Nevertheless the underlying causes of SB remain unknown and several conflicting hypotheses have been ventured regarding this issue [12], [19], [49], [50]. An entire study focusing on this complex phenomenon would be necessary to interpret this result with more reliability, but it was not our aim in the present study.

In terms of location in the cage, captive-born monkeys occupied more space as they were located more often in the upper, bottom and front parts of the cage while wild-born individuals stayed mainly on sitting benches. The location in the front of the cage is sometimes used as an indicator of tolerance of (or attraction to) the observer [51]. Therefore the captive-born individuals might be more tolerant to the observer’s presence than the wild-born monkeys. Another explanation could simply be the significantly higher levels of investigation and SB among captive-born animals. The more active individuals are, the lower the probability to be located at the resting spots gets.

Captive-born animals were interestingly standing at lower distances from one another compared to the wild-born monkeys. Some studies have shown that the strictness of the hierarchy impacts on the inter-individual distances, with monkeys from tolerant-hierarchical troops standing closer to their peers than monkeys from highly strict hierarchies [52], [53]. As hypothesized above, if the captive-born individuals expressed an altered/more permissive hierarchy, it might explain that they stand closer to each other compared to the wild-born groups.

The slumped body posture, characteristic of depressive-like individuals [54], was observed significantly more among wild-born animals. However whilst in this posture, both populations expressed a similar amount of inactivity. The identification of depressive-like animals using the indicator “inactive while slumped” [18] seems therefore appropriate only after hierarchical clustering when considering clusters’ rather than the total population’s mean occurrences.

To identify atypical profiles within populations, data were submitted to multiple component analyses (MCA) and hierarchical clustering. The first factorial plane of the MCA accounted for 24.3% or 22.3% of the total variance in the captive- and wild-born population respectively. These percentages are relatively low but likely explained by the important number of active modalities intentionally included in the analysis in order to prevent any “choice-bias”. They are also consistent with our previous study [18] considering the number of active modalities. The significant statistical differences between the clusters further establish the relevance of these descriptive analyses.

### Captive-born Animals

#### Seven profiles resulted from the hierarchical cluster analysis

A group (n = 1) was characterized by the highest level of displacement, feeding and social behaviours and the lowest proportion of inactivity. Displacement behaviours have been reported in stressful situations in both human and non-human populations [55], [56]. This individual could likely be at the bottom of the ranking hierarchy, thereby being more vigilant/stressed (explaining the low level of inactivity and high level of displacement behaviours) and having access to the food when the other members of the group were done eating (*i.e.* during the observational sessions that took place outside the feeding times). Due to the difficulty of assessing the intermediate hierarchical ranks among large groups of females (when all the subjects are not included in the observed sample), we could not collect this parameter. The detail of social behaviours however suggested that our hypothesis could be right since this monkey spent 23.1% of its time grooming peers and only 3.4% of its time being groomed. It has indeed been shown that subordinate macaques groom more than they are groomed, conversely to dominants [57].

B to F groups significantly differed on several collected variables (behaviours, postures, body orientations, locations and distances). These differences might be explained by distinct temperaments or personality traits, defined as factors influencing an individual’s perception of a situation and orchestrating its behavioural responses [58], and which have been suggested as determining causal factors for several social and emotional phenomena (*e.g.* dominance or response to an unfamiliar stimuli [59], [60]).

G group presented several similarities with the depressive single-housed male individuals from our previous study [18]: high levels of inactivity, and location in the back of the cage, and low levels of investigation, maintenance, and four-legged body posture. These features are in accordance with a few diagnostic criteria of a major depressive episode, described in the Diagnostic and Statistical Manual of Mental Disorders (DSM-IV), such as the decrease of interest or pleasure in usual activities, the psychomotor slowdown, and energy loss [17]. The slumped body posture, an acknowledged characteristic of depressed animals [54], is displayed by G group animals, similarly to B group. However, in opposition to B group, G animals were inactive 99.6% of the time spent in the slumped posture, supporting the idea of an indicator of poor well-being. The absence of significance between the “inactivity while slumped” percentages of B and G groups was likely a low group size effect (n_B_ = 3; n_G_ = 4). The mean behavioural diversity of the group was also the lowest of the captive-born individuals, though almost twice the diversity observed in the single-housed depressive males [18]. It is fully conceivable that more distinct behaviours can be expressed in a large enclosure and a social environment with individuals from both genders and diverse age categories compared to a single cage. We found contradictory results concerning feeding behaviours that were often expressed here and rarely displayed in our previous study. However major depressive disorder can be associated with either a loss or a gain of appetite according to the DSM-IV [17]. A lack of decreased feeding behaviours in cluster G was therefore not at odds with a depressive-like state. Although a decrease in social interactions could be expected from depressive-like individuals, we did not observe it. Recent studies however supported our findings by reporting no difference in the percentage of time spent groomed, more time spent in physical contact and less time spent alone in depressed cynomolgus macaques compared to non-depressed individuals [54], [57]. Further behavioural and neurobiological studies are needed to assess the consistency of our promising findings regarding this depressive-like profile.

### Wild-born Animals

H and I groups significantly differed on several parameters. Similarly to the captive-born B to F groups, the respective characteristics of these clusters are likely due to distinct personalities or social ranks. Every macaque troop functions following a relatively strict matrilineal hierarchy where dominants benefit from special privileges such as longer grooming session, easy access to food and to the best resting spots, and increased reproductive success [31], [38], [39]. H group might include more cautious, apprehensive or low ranking animals. Indeed, these individuals expressed more displacement (reflecting anxiety according to the literature [55], [56], [61] and as said in the previous paragraph), stereotypic and feeding behaviours than I group. The observational sessions took place outside the feeding times; therefore animals that ate during the scans were the last ones to access the food, namely subordinates. Conversely, I group might gather bold or high ranking individuals as they seemed to have priority access to food (thereby rarely eating during the scans), novelty (high level of object handling), and sitting benches. We did not highlight direct social differences although it has been shown that dominants sent more aggressive behaviours and are groomed more often than subordinates [57]. I group, however, spent significantly more time facing the open environment and less time facing peers compared to H group, which could reflect the “avoidance” of the congeners towards these potentially-high ranking individuals.

The only individual forming J group presented a profile mimicking some depressive-like symptoms, although caution must apply since this data could not be submitted to statistical tests. Similarly to G group and to the single-housed depressive-like individuals from our previous study [18], this monkey expressed a high level of inactivity, and low levels of locomotion, social and maintenance behaviours, resulting in a low behavioural diversity. Altogether, these features recall the decrease of interest or pleasure in usual activities, the psychomotor slowdown, and energy loss often seen in depressive patients [17] (see above for discussion of similar behaviours in captive-born animals). This female also spent much time located at the back of the cage and against a peer but unlike group G, it spent 99.6% of its time at the bottom of the cage and 80.7% of the time facing the wall, mainly in inactivity. In accordance with our previous paper [18], inactivity while facing a wall seems specific of a depressive-like state and to reflect a decreased interest toward the environment. Indeed unresponsiveness to environmental stimuli has recently been reported in “withdrawn” horses (characterized by atypical gaze, head and ears fixity), suggested as an ethological model of depression [15]. This individual might display an advanced depressive-like state compared to group G which did not face the wall as often.

Interestingly, the size of the depressive-like groups was 4 times higher in the captive-born population compared to the wild-born (4 vs 1 individuals respectively). Although we did not have much information concerning the wild-born first years of life, this finding supports the acknowledged concept that early adversity increases the predisposition to later-life behavioural abnormality [4]–[7] and especially the hypothesis of cumulative stress, *i.e.* the risk of pathology increases as adversity accumulates throughout life, in opposition to the mismatch effect (*i.e.* risk increases when the degree of mismatch between early- and later-life environments increases) [26], [44]. Nevertheless early life in a natural environment did not completely abolish the effects of suboptimal housing conditions at the adult age, since one individual displayed an atypical profile similar to a depressive-like state. Unexpectedly, the prevalence of depressive-like individuals among captive-born socially-housed females was similar to the one we found in singly-housed males [18]. We hypothesize an earlier onset of the atypical profile in singly-housed individuals (that were 3 years old compared to the 6 year-old individuals in this study) rather than deny the beneficial effect of social housing on macaques’ well being: more, or less, depressive-like individuals may have been identified, had we observed older singly-housed, or younger socially-housed macaques, respectively.

A final interesting point was the number of clusters resulting from hierarchical clustering in each population: 7 vs 3 in the captive- and wild-born group respectively. Such homogeneity in the wild-born population might again reflect a potentially more classic hierarchical organization, with a behavioural responses to captivity in relation to the individual rank (*i.e.* potentially-low and middle in cluster H or potentially-high in cluster I). Within their natural early life environment, these individuals acquired the appropriate social skills and learned the responses to an adverse event (*e.g.* presence of humans or other predators) from adults [40], [42]. On the contrary, captive-born monkeys were removed from a naturalistic social group before the wild weaning time (*i.e.* 6 months of age instead of 1 year old in the wild [22]–[24]), preventing a complete appropriate learning of the macaque social organization or appropriate coping mechanisms toward adversity. Several behavioural profiles might therefore emerge from this lack of wild-like social structure.

### Limitations

Along this discussion we have addressed a few points that need to be kept in mind when interpreting the differences between the captive- and wild-born populations. These parameters are for instance, but not limited to, age, maternal experience (*i.e.* the number of parturitions) and social ranks. The later element would be key in follow-up experiments and would require adaptations in the experimental design to collect this information. Although precautions have been taken with the carrying out of a habituation phase prior to the observations and the lack of significant differences in the level of behaviours directed towards the observer, we cannot rule out the possibility that wild-born individuals might be differentially affected by the observer’s presence compared to captive-born animals.

High-density housing conditions have been shown to modify animal behaviour [13], [62]. As the group sizes varied from 17 to 27 females, one could argue that the group size impacted the level of displacement behaviours instead of the origin of the animals. Positive Spearman correlations were indeed significant between group size and displacement behaviours, but were so in both the wild- and captive-born populations (Spearman rank order correlations: R_wild_ = 0.38 and R_captive_ = 0.42, p<0.05). High density by itself is therefore not sufficient to explain the increased level of displacement behaviours in the wild-born animals.

Finally, we availed of the presence of wild-born animals in the breeding facility to assess whether depressive-like individuals could also be identified in populations with a non-captive early life experience. Having little information about the history of the wild-born animals, our hypothesis regarding the likely protective effect of such naturalist experience is based on descriptive results. Quantifiable early life events would be required when investigating the impact of adversity on behaviours in order to establish any causal link between the 2 elements.

## Conclusions

Supporting our previous results in singly-housed males, inter-individual differences were also observed in spontaneous behaviours through unbiased ethological observations of socially-housed females. Several distinct behavioural profiles were identified including 2 promising clusters reminiscent of certain depressive-like symptoms. Four captive-born and one wild-born individuals indeed expressed such pathological profiles suggesting that natural social environment during infancy and youth does not prevent the development of abnormal behaviours in captive cynomolgus adults but aversive early life experience increases by 4 the risk of expressing them. Experimental research protocols should thus take into account the origin of the animals. The use of unbiased behavioural observations might allow the identification of animal models of human mental/behavioural disorders and their most appropriate control groups.

## Supporting Information

Figure S1 **Schematic plan of a common social-housing cage.** Monkeys were housed in indoors cages with opaque concrete walls on each side and at the back (clear parts). The floor, front and one part of the roof were wire meshed (grey parts). The cage’s measurements and virtual divisions (used to collect locations, see **Table S2**) are provided as follow: width (blue features), depth (red features) and height (green features). A swing was attached to the roof. Food was provided in a detachable feeding tray. Water was available *ad libitum* through 2 water pipes in the back of the cage.
(TIF)

Table S1 **Socially-housed cynomolgus monkey behavioural repertoire.** Collected detailed items (adapted from [18]) were then grouped for multiple component analysis (MCA).
(DOCX)

Table S2 **Locations, distances to nearest peer, body postures and orientations items displayed by socially-housed cynomolgus monkeys.** Collected detailed items (adapted from [18]) were then grouped for multiple component analysis (MCA). See **Figure S1** for visual support regarding the location parameters.
(DOCX)

Table S3 **Correlations between age or parturition number and other collected variables in captive- and wild-born populations.** A selection of Spearman rank order correlations between age or parturition number and other collected variables are presented below. These statistical analyses were performed separately in both populations. Significance (p<0.05) is indicated by bold numbers and a star (*).
(DOCX)
