# Supplementary figures and images for: Birth Origin Differentially Affects Depressive-Like Behaviours: Are Captive-Born Cynomolgus Monkeys More Vulnerable to Depression than Their Wild-Born Counterparts?
Source: PLoS One. 2013 Jul 4;8(7):e67711. doi: 10.1371/journal.pone.0067711 (PMC3701588; doi:10.1371/journal.pone.0067711)

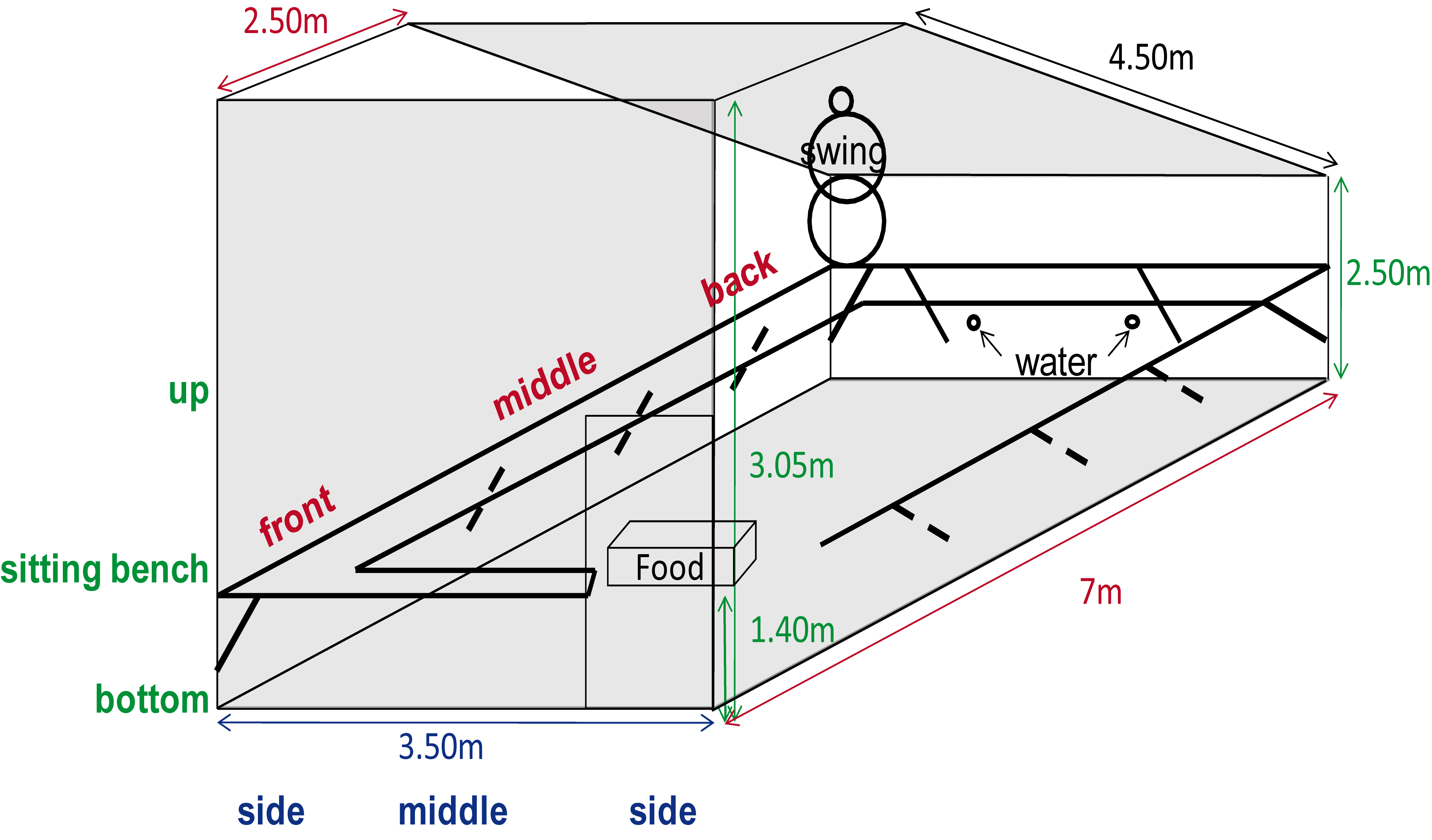

Supplement: Figure S1 — Schematic plan of a common social-housing cage. Monkeys were housed in indoors cages with opaque concrete walls on each side and at the back (clear parts). The floor, front and one part of the roof were wire meshed (grey parts). The cage’s measurements and virtual divisions (used to collect locations, see Table S2) are provided as follow: width (blue features), depth (red features) and height (green features). A swing was attached to the roof. Food was provided in a detachable feeding tray. Water was available ad libitum through 2 water pipes in the back of the cage. (TIF) [file pone.0067711.s001.tif]
