# Supplementary material for: Birth Origin Differentially Affects Depressive-Like Behaviours: Are Captive-Born Cynomolgus Monkeys More Vulnerable to Depression than Their Wild-Born Counterparts?
Source: PLoS One. 2013 Jul 4;8(7):e67711. doi: 10.1371/journal.pone.0067711 (PMC3701588; doi:10.1371/journal.pone.0067711)
Supplement: Table S1 — Socially-housed cynomolgus monkey behavioural repertoire. Collected detailed items (adapted from [18]) were then grouped for multiple component analysis (MCA). (DOCX) [file pone.0067711.s002.docx]

| **Grouped behaviours for MCA** | **Detailed collected behaviours** |
| --- | --- |
| **displacement behaviours** | scratches self |
|  | yawns |
|  | vacuum chew (chews despite empty mouth and cheekpouches) |
| **feeding** | "hunts" insect (try or manage to catch) |
|  | eats or drinks |
| **behaviours towards human** | interacts with observer (threat, submission, lipsmacking, genital display) |
| **inactivity** | immobility: not engaged in any other behaviours, with open eyes |
|  | rests: not engaged in any other behaviours, with closing or closed eyes |
|  | tensed inactivity (usually after an aggressive encounter) |
| **investigation** | manipulates collar with hands and/or mouth non-repetitively |
|  | manipulates toy with hands and/or mouth non-repetitively |
|  | manipulates other object with hands and/or mouth non-repetitively |
|  | cage investigation (searches, sniffs wall or bars) |
| **locomotion** | locomotion : change of location without any other behaviours |
|  | dangles on the swing |
| **maternal behaviours** | grooms infant or displays affiliative facial expression - positive |
|  | rejects, threatens, chases, bites or slapes infant - negative |
|  | retrieves or restraints infant - restrictive |
| **maintenance behaviours** | selfgrooms: grooming of own body |
|  | maintenance behaviours (urinate, defecate, rub eyes) |
|  | bites its nails |
|  | rubs hands on bars or floor |
| **social behaviours** | grooms peer- affiliative |
|  | is groomed by peer - affiliative |
|  | presents body part to be groomed - affiliative |
|  | presents genitals in a non-sexual context - affiliative |
|  | lipsmacks - affiliative |
|  | other affiliative behaviours (facial expression, seak positive contact with peer) |
|  | threatens, chases, bites or slapes peer - agonistic |
|  | flees or displays submissive facial expression - submissive |
| **sexual behaviours** | typical sexual behaviour (presents genitals to male, mates) |
| **vocalization** | vocalizes |
| **stereotypic behaviours** | manipulates collar repetitively - manual |
|  | manipulates other object repetitively - manual |
|  | picks peer’s or own fur repetitively and outside grooming context - manual |
|  | motor stereotypy (pacing, flipping, walks backwards,…) - motor |
|  | gnaws bars repetitively - oral |
|  | licks bars repetitively - oral |
|  | licks own tail or other body part repetitively - oral |
|  | bites own tail or other body part repetitively - oral |
|  | oral stereotypy (tongue movement or tongue chew) - oral |
|  | kidnapping: steals infant from its mother |
|  | self suckling |
