# Supplementary material for: Birth Origin Differentially Affects Depressive-Like Behaviours: Are Captive-Born Cynomolgus Monkeys More Vulnerable to Depression than Their Wild-Born Counterparts?
Source: PLoS One. 2013 Jul 4;8(7):e67711. doi: 10.1371/journal.pone.0067711 (PMC3701588; doi:10.1371/journal.pone.0067711)
Supplement: Table S2 — Locations, distances to nearest peer, body postures and orientations items displayed by socially-housed cynomolgus monkeys. Collected detailed items (adapted from [18]) were then grouped for multiple component analysis (MCA). See Figure S1 for visual support regarding the location parameters. (DOCX) [file pone.0067711.s003.docx]

| **Variables for MCA** | **Detailed collected variables** |
| --- | --- |
| **Body postures** |  |
| **seated** | resting on the buttocks with straight back |
|  | resting on the buttocks with bent back |
|  | resting on the buttocks with stretched legs |
| **biped** | standing on hind limbs (biped) |
| **slumped** | slumped (seated head lower than shoulder's line) |
| **lying down** | lying down |
| **on bars** | seated posture but on a wire mesh part of the cage |
|  | upside down four-legged (hanging on wire meshed ceiling) |
|  | suspending in any other way (four limbs on a wire meshed part of the cage) |
| **four-legged** | four-legged, hanging tail |
|  | four-legged, tail in "?" shape |
|  | four-legged, tail above head |
|  | four-legged, straight tail (in the continuity of the back) |
|  | « bottom up » (four-legged with head and shoulders on the ground level) |
|  | crouched (ventral surface close to floor; head at or below the level of the shoulders) |
|  |  |
| **Body orientations** |  |
| **peer** | body oriented toward peer |
| **exterior** | body oriented toward exterior/observer |
| **ground** | body oriented toward ground or ceiling |
| **wall** | body oriented toward a wall of the cage (max. 50 cm from wall) |
| **open environment** | body oriented toward open environment (none of the above) |
|  |  |
| **Locations in cage** |  |
| **side** | cage width divided in 3 virtual parts: side or middle |
| **middle** |  |
| **front** | cage depth divided in 3 virtual parts: front, middle or back |
| **middle** |  |
| **back** |  |
| **bottom** | cage height divided in 3 virtual parts: up, sitting bench or bottom |
| **sitting bench** |  |
| **up** |  |
|  |  |
| **Distance to nearest peer** |  |
| **against** | body in direct physical contact with a peer |
| **d. < 1arm** | within 1 arm from peer |
| **1arm<d.<1m** | between 1 arm and 1 meter from peer |
| **1m<d.<3m** | between 1 meter and 3 meter from peer |
| **d.>3m** | more than 3 meter from peer |
