# Supplementary material for: Birth Origin Differentially Affects Depressive-Like Behaviours: Are Captive-Born Cynomolgus Monkeys More Vulnerable to Depression than Their Wild-Born Counterparts?
Source: PLoS One. 2013 Jul 4;8(7):e67711. doi: 10.1371/journal.pone.0067711 (PMC3701588; doi:10.1371/journal.pone.0067711)
Supplement: Table S3 — Correlations between age or parturition number and other collected variables in captive- and wild-born populations. A selection of Spearman rank order correlations between age or parturition number and other collected variables are presented below. These statistical analyses were performed separately in both populations. Significance (p<0.05) is indicated by bold numbers and a star (*). (DOCX) [file pone.0067711.s004.docx]

| **R_S_ per variable** | **Captive-born** | | **Wild-born** | |
| --- | --- | --- | --- | --- |
|  | **Age** | **Parturition**  **number** | **Age** | **Parturition**  **number** |
| **parturition number** | 0.209 | **-** | 0.238 | - |
| ***Behaviours:*** |  |  |  |  |
| displacement B.: | -0.109 | -0.247 | 0.051 | 0.160 |
| scratch | -0.197 | -0.020 | 0.042 | 0.203 |
| vacuous chew | -0.138 | -0.265 | 0.094 | 0.114 |
| yawn | 0.214 | -0.056 | -0.184 | -0.172 |
| feeding B. | -0.152 | **-0.483 *** | -0.295 | -0.048 |
| B. toward human | 0.152 | 0.203 | 0.048 | 0.108 |
| inactivity: | **0.398 *** | 0.133 | -0.027 | -0.270 |
| immobility | 0.253 | 0.132 | -0.083 | -0.270 |
| resting B. | 0.225 | -0.042 | 0.109 | 0.131 |
| investigation: | -0.236 | -0.214 | **-0.358 *** | -0.139 |
| play B. | -0.135 | 0.122 | -0.094 | 0.026 |
| locomotion | -0.218 | 0.240 | 0.208 | 0.271 |
| maternal B. | 0.033 | **0.687 *** | 0.009 | 0.236 |
| maintenance B. | -0.008 | **-0.386 *** | 0.015 | 0.236 |
| social B.: | 0.023 | -0.175 | 0.248 | 0.215 |
| agonistic B. | 0.059 | **0.399 *** | 0.297 | 0.250 |
| stereotypic B. | -0.310 | 0.207 | -0.107 | -0.108 |
| manual SB. | 0.070 | **0.334 *** | 0.081 | 0.045 |
| motor SB. | -0.282 | -0.023 | -0.094 | 0.026 |
| oral SB. | **-0.456 *** | 0.076 | **-0.324 *** | -0.200 |
| **Behavioural diversity** | -0.170 | **0.333 *** | -0.172 | 0.244 |
| ***Body postures:*** |  |  |  |  |
| seated | **0.545 *** | -0.074 | -0.051 | -0.270 |
| biped | -0.113 | 0.072 | -0.047 | 0.114 |
| slumped | **-0.320 *** | -0.030 | -0.016 | 0.062 |
| lying down | -0.109 | -0.269 | 0.199 | 0.104 |
| on bars | **-0.343 *** | -0.261 | 0.005 | 0.172 |
| four-legged: | -0.308 | 0.164 | 0.023 | 0.257 |
| crouched | -0.276 | **-0.340 *** | -0.156 | -0.213 |
| "bottom up" | -0.111 | -0.058 | -0.208 | -0.190 |
| ***Behaviours while slumped*** |  |  |  |  |
| displacement B. | -0.172 | 0.237 | 0.118 | 0.047 |
| inactivity | 0.106 | 0.023 | -0.062 | -0.072 |
| maternal B. | -0.028 | **0.423 *** | -0.008 | 0.135 |
| maintenance B. | **-0.374 *** | -0.108 | -0.029 | 0.129 |
| social B. | -0.010 | -0.043 | 0.116 | -0.044 |
| ***Body orientations:*** |  |  |  |  |
| peer | -0.023 | -0.072 | 0.087 | 0.270 |
| exterior | 0.098 | 0.047 | 0.057 | 0.243 |
| ground | **0.337 *** | 0.062 | 0.118 | 0.243 |
| wall | -0.272 | -0.217 | -0.116 | -0.270 |
| open environment | 0.107 | 0.105 | -0.036 | 0.270 |
| ***Behaviours while facing wall*** |  |  |  |  |
| feeding B. | -0.045 | **-0.392 *** | 0.025 | 0.116 |
| inactivity | 0.169 | 0.092 | 0.189 | -0.275 |
| investigation | -0.222 | -0.096 | -0.033 | -0.082 |
| maintenance B. | -0.132 | -0.279 | -0.051 | -0.031 |
| social B. | 0.288 | -0.120 | 0.117 | 0.059 |
| stereotypic B. | -0.066 | 0.104 | -0.235 | -0.245 |
| ***Locations in the cage:*** |  |  |  |  |
| front | -0.201 | 0.141 | -0.197 | 0.215 |
| back | 0.133 | -0.004 | 0.232 | -0.270 |
| bottom | -0.281 | -0.250 | **-0.434 *** | -0.270 |
| sitting bench | 0.307 | 0.303 | **0.429 *** | 0.270 |
| up | **-0.337 *** | -0.234 | -0.045 | 0.166 |
| ***Distances to nearest peer:*** |  |  |  |  |
| against | 0.281 | 0.330 | -0.036 | -0.270 |
| d. < 1arm | -0.115 | -0.099 | 0.220 | 0.270 |
| 1arm<d.<1m | -0.153 | -0.168 | 0.043 | 0.270 |
| 1m<d.<3m | **-0.382 *** | **-0.396 *** | -0.026 | 0.132 |
| d.>3m | **-0.343 *** | **-0.362 *** | -0.113 | -0.244 |
